# Simultaneous Immunoglobulin G4-associated Autoimmune Hepatitis and Autoimmune Pancreatitis

**DOI:** 10.5005/jp-journals-10018-1223

**Published:** 2017-05-05

**Authors:** Birnur Yilmaz, Safak Kiziltas, Semsi Yildiz, Burçak Gümüs, Halime Çevik

**Affiliations:** 1 Department of Radiology, Faculty of Medicine, Okan University, Istanbul, Turkey; 2 Department of Gastroenterology, Baskent University Istanbul, Turkey; 3 Department of Pathology, Baskent University, Istanbul, Turkey; 4 Department of Radiology, Baskent University, Istanbul Turkey

**Keywords:** Autoimmune hepatitis, Autoimmune pancreatitis, Immunoglobulin G4.

## Abstract

**How to cite this article:** Yilmaz B, Kiziltas S, Yildiz S, Gümüs B, Çevik H. Simultaneous Immunoglobulin G4-associated Autoimmune Hepatitis and Autoimmune Pancreatitis. Euroasian J Hepato-Gastroenterol 2017;7(1):95-96.

Dear Editor,

The case of a patient with immunoglobulin G4 (IgG4)-associated autoimmune hepatitis (AIH) and autoimmune pancreatitis (AIP) is presented. With increased levels of serum IgG4 and abundant IgG4 positive plasma cell infiltration, AIH was reported and proposed to be called "IgG4-associated AIH." However, the clinical course of this new disease entity remains unclear. A 57-year-old woman presented at our institution with weakness, sickness, darkening of urine color. Patient was admitted to our hospital due to elevated levels of serum aspartate aminotransferase, alanine aminotransferase, gamma-glutamyltransferase, bilirubin levels, CA 19-9, amylase, and lipase. Prothrombin time was prolonged. Alfa-fetoprotein levels were normal. The IgG4 level of 5,050 mg/dL was detected. Antinuclear antibody was positive; antismooth muscle antibody and antimitochondrial antibody were negative. Hepatitis B virus deoxyribonucleic acid (-), Hepatitis C virus ribonucleic acid (-), and anti-hepatitis A virus IgG (+) were detected. Patients had never used alcohol. In conclusion, except for high serum IgG4 levels and IgG4 positive plasma cell infiltration, the findings in patients with IgG4-associated AIH are consistent with the findings in patients with classical AIH. Therefore, there may be many more patients than formerly thought who are diagnosed as having classical AIH, but who actually have IgG4-associated AIH. Further cases need to be evaluated to clarify the clinical course and the standard treatment for IgG4-associated simultaneous AIH and AIP.

## INTRODUCTION

A rare case with immunoglobulin G4 (IgG4)-associated autoimmune hepatitis (AIH) and autoimmune pancreatitis (AIP) is presented. Autoimmune hepatitis is characterized by increased levels of serum (IgG4) and abundant IgG4-positive plasma cell infiltration and proposed to be called "IgG4-associated AIH."^1^ The IgG4-associated diseases include pancreatitis and cholangitis. The IgG4-associated AIH has also been proposed, but whether it represents a distinct entity is not yet clearly defined.^2^ Autoimmune pancreatitis is described by high serum IgG4 levels and lymphoplasmacytic inflammation.^3^

## CASE REPORT

**Fig. 1:**
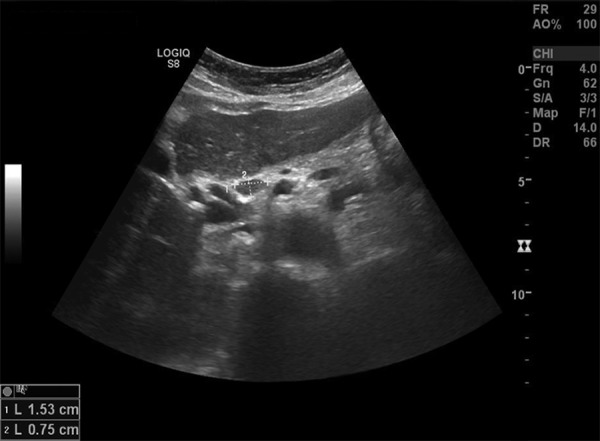
Ultrasonography of abdomen

**Fig. 2:**
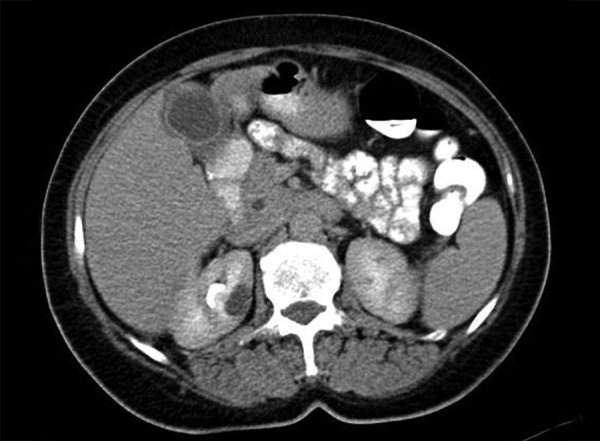
Magnetic resonance imaging of abdomen

A 57-year-old woman presented at our institution with weakness, sickness, darkening of urine color. Patient was hospitalized with increased levels of liver transaminases, gamma-glutamyltransferase, bilirubin, amylase, and lipase. Aspartate aminotransferase levels were 1,369 U/L and alanine aminotransferase levels were 1,120 U/L. Gamma-glutamyltranspeptidase of 596 U/L, amylase 363 U/L, lipase 622 U/L, CA 19-9: 715 IU/mL, and IgG4 5050 mg/dL were detected. Antinuclear antibody was high titer at 1:320. Hepatitis markers were negative. The patient provided a written consent form. Granula echogenicity in the liver, increase in the wall thickness of gallbladder, peripancreatic lymphadenopathy were detected on abdominal ultrasound examination. Computerized tomography demonstrated the same findings as ultrasound examination (Figs 1 and 2).

**Fig. 3:**
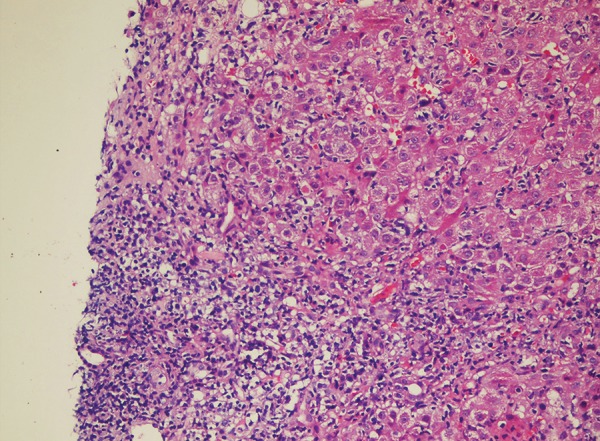
Histopathological finding of the liver

Magnetic resonance cholangiopancreatography was normal. Liver biopsy findings are not specifically diagnostic. In general, the biopsy proved the presence of chronic hepatitis and demonstrated no clue for the discrimination of other causes of chronic hepatitis. Typically, the biopsy shows an irregularly distributed relatively heavy portal infiltrate, with periportal or paraseptal interface hepatitis with increased numbers of plasma cells and eosinophils in addition to lymphocytes. In liver biopsy interphase activity, confluent necrosis, hepatic duct proliferation, and biliary stasis were detected (Fig. 3). Pancreas fine-needle biopsy was performed, but no additional finding was observed. The AIH diagnosis was made according to the International Autoimmune Hepatitis Group score of 18, indicative of "definite AIH." Prednisolone (60 mg/day) was started, and liver transaminases decreased rapidly.

## DISCUSSION

Our case clearly demonstrates that IgG4-associated AIH and AIP can be simultaneously present as part of a clinical spectrum. This clinical situation should be considered as a possible diagnosis in patients with classically diagnosed AIH. The publication of more cases of IgG4-associated simultaneous AIH and AIP will determine the clinical importance and prevalence of this uncommon entity.
